# MicroRNA-27b inhibits Spry2 expression and promotes cell invasion in glioma U251 cells

**DOI:** 10.3892/ol.2015.2865

**Published:** 2015-01-12

**Authors:** CHENGHUI LIU, SHIXING LIANG, SHENGHUI XIAO, QIMING LIN, XU CHEN, YI WU, JIAN FU

**Affiliations:** Department of Neurosurgery, Nanhai Hospital of Southern Medical University, Foshan, Guangdong 528200, P.R. China

**Keywords:** glioma, microRNA-27b, sprouty homolog 2, invasion

## Abstract

MicroRNA (miR)-27b has been reported to participate in glioma. However, a detailed role of miR-27b and the underlying mechanism remain largely unknown. The present study found that the expression of miR-27b was significantly increased in glioma tissues compared with normal adjacent tissues. In addition, miR-27b was also upregulated in the U87, U251 and SHG44 glioma cell lines compared with normal human astrocytes. Sprouty homolog 2 (Spry2), which has been reported to be associated with invasive glioma, was identified as a novel target of miR-27b in U251 glioma cells, and the protein expression of Spry2 was negatively regulated by miR-27b in U251 cells. Additionally, inhibition of miR-27b and upregulation of Spry2 suppressed glioma cell invasion, while downregulation of Spry2 reversed the suppressive effect of miR-27b inhibition on glioma cell invasion. These data suggest that miR-27b may promote glioma cell invasion through direct inhibition of Spry2 expression. The data also suggest that miR-27b may become a promising molecular target for inhibiting the invasion and metastasis of glioma.

## Introduction

Glioma is among the most common human malignancies in the brain. Although the five-year survival rate of patients with glioma has been improved in recent years due to the combination of surgery, radiotherapy and chemotherapy, the prognosis of patients with invasive glioma remains poor (1,2). It has been demonstrated that dysfunction of oncogenes or tumor suppressors is closely associated with the development and progression of glioma (2). Accordingly, developing novel molecular targets may be promising for the development of therapeutic strategies for invasive glioma.

MicroRNAs (miRNAs), a class of non-coding RNAs 18–25 nucleotides in length, can induce mRNA degradation or suppress protein translation by binding to the seed sequences in the 3′-untranslational region (UTR) of mRNAs. By negatively regulating the protein expression of their targets, microRNAs exert adverse effects on cell survival, proliferation and motility (3). Dysfunctions of miRNAs, which act as oncogenes or tumor suppressors, have been demonstrated to be associated with human malignancies (4). Furthermore, deregulations of numerous miRNAs have been revealed to contribute to the development and progression of invasive glioma, including miRNA-20a (miR-20a), miR-106a, miR-145, miR-494 and miR-124 (5–8). MiR-27b has been reported to be associated with glioma (9). However, the detailed role of miR-27b in the regulation of invasive glioma remains largely unknown.

Sprouty homolog 2 (Spry2), a member of the sprouty family, contains a carboxyl-terminal cysteine-rich domain essential for the inhibition of receptor tyrosine kinase signaling (10) Spry2 can function as a regulator of mitogen-activated protein kinase signaling, which plays a crucial role in the regulation of cancer cell invasion (11,12). The protein level of Spry2 has previously been reported to be significantly decreased in invasive glioma tissues, suggesting that Spry2 may participate in the regulation of glioma invasion (13). However, the underlying molecular mechanism remains unclear.

The present study aimed to explore the roles of miR-27b and Spry2 in the regulation of glioma cell invasion. In addition, the underlying molecular mechanism of these roles was investigated.

## Materials and methods

### Tissue specimen collection

The present study was approved by the Ethical Committee of Nanhai Hospital of Southern Medical University (Foshan, China). In total, 30 glioma tissue and matched adjacent normal tissue samples were obtained from the Department of Neurosurgery of Nanhai Hospital of Southern Medical University. For each patient, informed consent was obtained. Subsequent to surgical removal, the tissue samples were frozen in liquid nitrogen until usage.

### Cell culture

The human glioma U87, U251 and SHG44 cell lines were purchased from the Cell Bank of Southern Medical University (Guangzhou, China). The human astrocyte HA cell line was purchased from ScienCell Research Laboratories (Carlsbad, CA, USA). The cells were cultured in Dulbecco’s modified Eagle’s medium (DMEM) with 10% fetal bovine serum (FBS) at 37°C in a 5 % CO_2_ atmosphere.

### Reverse transcription-quantitative polymerase chain reaction (RT-qPCR) assay

Total RNA was extracted using TRIzol reagent (Life Technologies, Carlsbad, CA, USA). An miRNA reverse transcription kit (Life Technologies) was used to convert RNA into cDNA, according to the manufacturer’s instructions. qPCR was then performed using an miRNA Q-PCR Detection kit (GeneCopoeia, Rockville, MD, USA) on the ABI 7500 thermocycler (Applied Biosystems Life Technologies, Foster City, CA, US). The U6 gene was used as an internal reference. Relative expression was analyzed by the 2^−ΔΔCt^ method.

### Western blotting

Tissues and cells were lysed in cold radioimmunoprecipitation assay lysis buffer. The proteins were separated with 10% SDS-PAGE, and transferred onto a polyvinylidene difluoride (PVDF) membrane. The PVDF membrane was incubated overnight with phosphate-buffered saline containing 5% milk at 4°C. Following incubation, the PVDF membrane was incubated with monoclonal mouse anti-human Spry2 (1:200; ab60719) and monoclonal mouse anti-human GAPDH primary antibodies (1:200; ab125247; Abcam, Cambridge, UK) at room temperature for 3 h, and then incubated with polyclonal rabbit anti-mouse secondary antibodies (1:5,000; ab175743; Abcam) at room temperature for 1 h. An electrochemiluminescence kit (Pierce Chemical, Rockford, IL, USA) was then used to perform chemiluminescent detection. The relative protein expression was analyzed by Image-Pro plus software 6.0 (Media Cybernetics, Inc., Rockville, MD, USA) and was presented as the density ratio versus GAPDH.

### Transfection

The cells were cultured to 70% confluence, and resuspended in serum-free medium. Lipofectamine 2000 (Life Technologies) was used to perform transfection according to the manufacturer’s instructions. Briefly, miR-27b inhibitor, Spry2 plasmid or Spry2 siRNA (all from Niunbio Company, Changsha, China) were diluted (1:50) with serum-free medium. Lipofectamine 2000 was also diluted (1:50) with serum-free medium. The diluted Lipofectamine 2000 was added to the diluted miR-27b inhibitor, Spry2 plasmid or Spry2 siRNA, incubated for 20 min at room temperature and then added to the cell suspension. The cells were then incubated at 37°C in 5% CO_2_ for 6 h. Following incubation, the medium in each well was replaced by normal serum-containing medium and cultured for 24 h prior to the subsequent assays. Bioinformatic analysis was performed to predicate the target association between miR-27b and Spry2.

### Dual luciferase reporter assay

A Directed Mutagenesis kit (Stratagene Inc., La Jolla, CA, USA) was used to generate a mutant type 3′-UTR of Spry2. The wild or mutant type 3′-UTR of Spry2 was inserted into the psiCHECK™2 vector (Promega, Madison, WI, USA). The U251 cells were transfected with the psiCHECK2-Spry2-3′-UTR or psiCHECK2-mutant Spry2 -3′-UTR vector, with or without 100 nM miR-27b mimics. Subsequently, the U251 cells were incubated at 37°C with 5 % CO_2_ for 48 h. The luciferase activities were then examined on a LD400 luminometer (Beckman Coulter, Brea, CA, USA). Renilla luciferase activity was normalized to firefly luciferase activity.

### Transwell invasion assay

A cell suspension containing 5×10^5^ cells/ml was prepared in serum-free media for the Transwell invasion assay (Corning Inc., Corning, NY, USA). Then, 500 μl of DMEM supplemented with 10% FBS was added into the lower chamber and 300 μl of the cell suspension was added to the upper chamber. The cells were then incubated at 37°C with 5 % CO_2_ for 24 h. Following incubation, the cells on the upper surface were removed using a cotton-tipped swab, while the cells on the lower surface were stained for 30 min. Under the microscope, the cell number was counted in at least five randomly selected fields.

### Statistical analysis

All data were expressed as the mean ± standard deviation. SPSS 17.0 software (SPSS, Inc., Chicago, IL, USA) was used to perform the statistical analysis. Differences were analyzed using one-way analysis of variance and P<0.05 was considered to indicate a statistically significant difference.

## Results

### MiR-27b was upregulated in glioma tissues and cell lines

**Figure 1:**
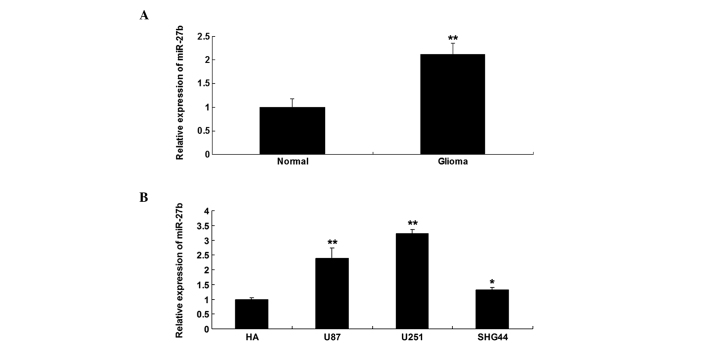
(A) The relative expression of miR-27b was examined by performing RT-qPCR in glioma tissues (Glioma) and their matched adjacent normal tissues (Normal). ^**^P<0.01, vs. Normal. (B) The relative expression of miR-27b was examined by performing reverse transcription-quantitative polymerase chain reaction in the human glioma U87, U251 and SHG44 cell lines as well as in the human astrocyte HA cell line. ^*^P<0.05 and ^**^P<0.01, vs. HA. miR, microRNA.

The expression level of miR-27b was determined using RT-qPCR in glioma tissues and their matched normal adjacent tissues. It was found that miR-27b was significantly upregulated in glioma tissues, when compared with normal adjacent tissues (Fig. 1A). The expression level of miR-27b was also determined in three common glioma cell lines, U87, U251 and SHG44, and in the normal human astrocyte HA cell line. Compared with normal human HA astrocytes, the glioma cells demonstrated notable upregulation of miR-27b, particularly in U251 cells (Fig. 1B). Accordingly, U251 cells were used in the subsequent experiments.

### Spry2 was identified as a direct target of miR-27b in U251 cells

**Figure 2:**
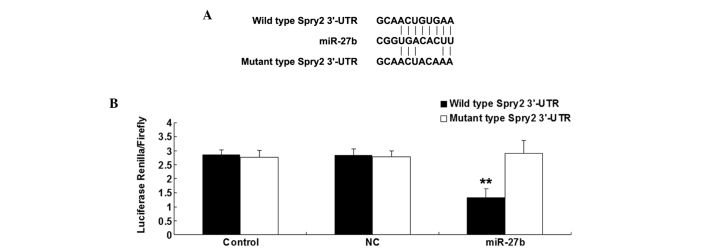
(A) The seed sequences of miR-27b in the wild or mutant type 3′-UTR of Spry2. (B) Luciferase reporter assay data found that co-transfection of U251 cells with miR-27b and wild type Spry2 3′-UTR led to a decrease in luciferase activity. However, co-transfection with mutant Spry2 3′-UTR and miR-27b mimics exhibited no effect. ^**^P<0.01, vs. Control. miR, microRNA; Spry2, Sprouty homolog 2; 3′-UTR, 3′-untranslational region; Control, cells co-transfected with blank vector and wild type or mutant Spry2 3′-UTR; NC, U251 cells transfected with microRNA scramble control; miR27b, cells transfected with miR-27b mimics.

Bioinformatic analysis was performed to predicate the target association between miR-27b and Spry2. The putative seed sequences for miR-27b in the 3′UTR of Spry2 were evolutionarily conserved. As shown in Fig. 2A, the wild and mutant types of the Spry2 3′-UTR were then generated. Subsequently, U251 cells were transfected with psiCHECK2-Spry2-3′-UTR or psiCHECK2-mutant Spry2 -3′-UTR vector, with or without miR-27b mimics. Following incubation for 48 h, the luciferase activity was examined. The present data revealed that the luciferase activity was notably reduced only in U251 cells co-transfected with the wild type 3′-UTR of Spry2 and miR-27b mimics, suggesting that miR-27b could directly bind to the 3′UTR of Spry2 mRNA in U251 cells (Fig. 2B).

### MiR-27b negatively regulated Spry2 expression at a post-transcriptional level in U251 cells

**Figure 3:**
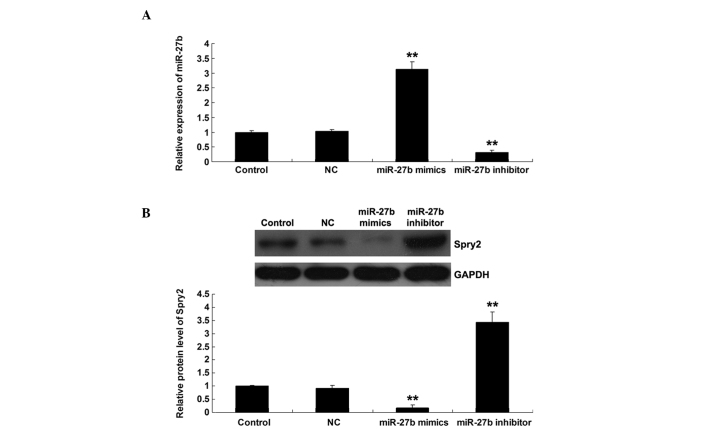
(A) The relative expression of miR-27b was determined in U251 cells transfected with miR-27b mimics or the miR-27b inhibitor and NC cells. (B) The protein level of Spry2 was examined in U251 cells transfected with miR-27b mimics or the miR-27b inhibitor and NC cells. ^**^P<0.01, vs. Control. miR, microRNA; NC, cells transfected with miRNA scramble control; control, U251 cells without any transfection; Spry2, Sprouty homolog 2.

U251 cells were transfected with scramble miRNA control (NC), miR-27b mimics or the miR-27b inhibitor. Subsequently, the expression level of miR-27b in each group was examined. As shown in Fig. 3A, the miR-27b level was significantly upregulated following transfection with miR-27b mimics, while notably reduced subsequent to transfection with the miR-27b inhibitor. To explore the regulatory association between miR-27b and Spry2, the protein level of Spry2 was determined in U251 cells transfected with the miR-27b mimics or inhibitor. As shown in Fig. 3B, upregulation of miR-27b significantly inhibited the protein expression of Spry2 in U251 cells. By contrast, inhibition of miR-27b resulted in an increased protein expression of Spry2 in U251 cells. Based on these data, it was suggested that miR-27b negatively regulated Spry2 expression at a post-transcriptional level in glioma U251 cells.

### MiR-27b promoted U251 cell invasion by targeting Spry2

**Figure 4:**
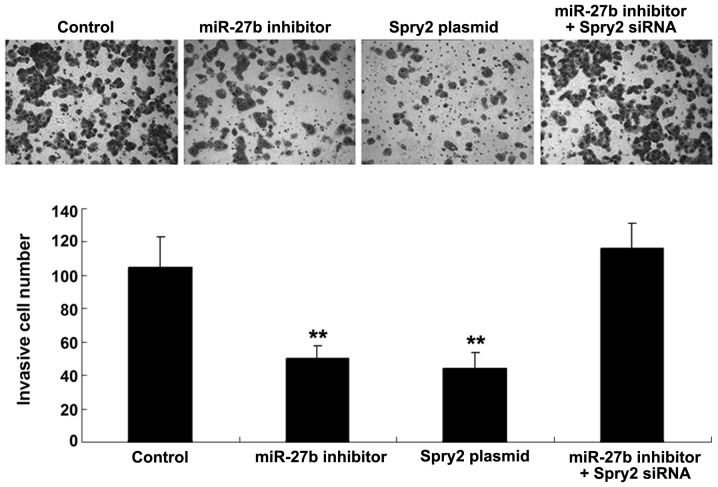
The invasion capacity was determined using Transwell assay in U251 cells transfected with the miR-27b inhibitor, Spry2 plasmid, or co-transfected with miR-27b inhibitor and Spry2 siRNA. ^**^P<0.01, vs. Control. Spry2, Sprouty homolog 2; siRNA, small interfering RNA; Control, U251 cells without any transfection; miR, microRNA.

As Spry2 has previously been suggested to be associated with invasive glioma (9) and miR-251 negatively regulated the Spry2 expression in U251 cells, it was speculated that miR-27b may also participate in the regulation of glioma U251 cell invasion. To verify this speculation, U251 cells were transfected with the miR-27b inhibitor or Spry2 plasmid, or the cells were co-transfected with the miR-27b inhibitor and Spry2 siRNA. It was then demonstrated that the inhibition of miR-27b activation and overexpression of Spry2 significantly suppressed U251 cell invasion (Fig. 4). However, the inhibitory effect of miR-27b inhibition on U251 cell invasion was attenuated by siRNA-induced Spry2 downregulation (Fig. 4). The present data suggested that miR-27b may promote the regulation of U251 cell invasion via direct targeting of Spry2.

## Discussion

Dysfunction of miR-27b and Spry2 has been found to be involved in the development and progression of glioma (9,13). However, to the best of our knowledge, the roles of and association between miR-27b and Spry2 have never been studied in glioma. In the present study, it was revealed that the expression level of miR-27b was markedly increased in glioma tissues and the U87, U251 and SHG44 glioma cell lines compared with normal brain tissue and astrocytes. In addition, the present study identified Spry2 as a direct target of miR-27b, and demonstrated that the protein expression of Spry2 was negatively regulated by miR-27b in glioma U251 cells. Furthermore, inhibition of miR-27b and upregulation of Spry2 could suppress glioma cell invasion, while downregulation of Spry2 reversed the suppressive effect of miR-27b inhibition on glioma cell invasion. Based on these data, it can be suggested that miR-27b may promote glioma cell invasion through direct inhibition of Spry2 expression.

miRNAs have been found to regulate various biological processes, and deregulation of miRNAs participates in the development and progression of human malignancies (14). The roles of numerous miRNAs in glioma have been widely investigated, including miR-20a, miR-106a, miR-145, miR-494 and miR-124 (5–8). MiR-27b has been demonstrated to be involved in several cancers. Wan *et al* revealed that miR-27b was notably decreased in non-small cell lung cancer (NSCLC) tissues and cell lines, and that overexpression of miR-27b significantly suppressed NSCLC cell proliferation and invasion (15), indicating that miR-27b acts as a tumor suppressor in NSCLC. The majority of studies have demonstrated that miR-27b plays an inhibitory role in the development and progression of human malignancies, including colon and prostate cancer and neuroblastoma (16–18). However, a few studies have also suggested that miR-27b may act as a tumor promoter. Jin *et al* revealed that miR-27b was highly upregulated in human breast cancer, and that knockdown of miR-27b substantially repressed breast cancer growth (19).

Chen *et al* previously suggested that miR-27b acts as an oncogene in glioma. This study found that miR-27b was upregulated in glioma tissues and cells (9), which is consistent with the present findings. However, the effect of miR-27b on glioma cell invasion and the involved mechanism remains largely unknown. In the present study, it was found that miR-27b played a promoting role in the regulation of glioma U251 cell invasion, and further molecular mechanism investigation suggested that the promotion of U251 cell invasion by miR-27b occurred partially by direct inhibition of Spry2.

As a negative regulator of receptor tyrosine kinase-mediated signaling, Spry2 has been found to play a role in various cancers (13,20,21). Spry2 mainly acts as a tumor suppressor. Rathmanner *et al* revealed that Spry2 inhibited cell proliferation and migration in osteosarcoma cells (21). Li *et al* reported that Spry2 was downregulated in renal cell carcinoma (RCC) tissues compared with adjacent normal tissues, and that Spry2 could inhibit RCC cell proliferation and invasion (20). Spry2 has previously been reported to be significantly downregulated in invasive glioma tissues, suggesting that Spry2 may participate in the regulation of glioma invasion (13). In the present study, it was revealed that overexpression of Spry2 significantly inhibited the invasion of glioma U251 cells. These findings suggest that Spry2 inhibits the regulation of glioma cell invasion. In addition, miR-27b was shown to regulate the protein expression of Spry2 by directly targeting the 3′UTR of Spry2 mRNA in glioma U251 cells. miR-27b has previously been reported to directly target Spry2 in zebrafish (22). However, the existence of this targeting association between miR-27b and Spry2 has never been reported in humans. Furthermore, by the gain of function assay, it was found that miR-27b inhibition led to a significant inhibition of U251 cell invasion, similar to the effect of Spry2 overexpression. Additionally, inhibition of Spry2 reversed the suppressive effect of miR-27b downregulation on glioma U251 cell invasion. These findings further confirmed that miR-27b plays a role in the regulation of glioma cell invasion through direct targeting of Spry2.

In conclusion, the present study suggests that upregulation of miR-27b in glioma may promote glioma cell invasion by inhibiting the expression of its target, Spry2. Therefore, miR-27b may serve as a promising target for the prevention and treatment of glioma invasion.

## References

[1] Watts C, Price SJ, Santarius T (2014). Current concepts in the surgical management of glioma patients. Clin Oncol (R Coll Radiol).

[2] Haapasalo J, Hyartt A, Salmi M (2014). Diagnosis and prognosis of gliomas - current prospects of molecular diagnostics. Duodecim.

[3] Peng Y, Yu S, Li H (2014). MicroRNAs: emerging roles in adipogenesis and obesity. Cell Signal.

[4] Palumbo S, Miracco C, Pirtoli L, Comincini S (2014). Emerging roles of microRNA in modulating cell-death processes in malignant glioma. J Cell Physiol.

[5] Wang Z, Wang B, Shi Y (2014). Oncogenic miR-20a and miR-106a enhance the invasiveness of human glioma stem cells by directly targeting TIMP-2. Oncogene.

[6] Wan X, Cheng Q, Peng R (2014). ROCK1, a novel target of miR-145, promotes glioma cell invasion. Mol Med Rep.

[7] Kwak SY, Yang JS, Kim BY, Bae IH, Han YH (2014). Ionizing radiation-inducible miR-494 promotes glioma cell invasion through EGFR stabilization by targeting p190B rhoGAP. Biochim Biophys Acta.

[8] An L, Liu Y, Wu A, Guan Y (2013). microRNA-124 inhibits migration and invasion by down-regulating ROCK1 in glioma. PLoS One.

[9] Chen L, Li H, Han L (2011). Expression and function of miR-27b in human glioma. Oncol Rep.

[10] Cabrita MA, Christofori G (2008). Sprouty proteins, masterminds of receptor tyrosine kinase signaling. Angiogenesis.

[11] Mei Y, Bian C, Li J (2013). miR-21 modulates the ERK-MAPK signaling pathway by regulating SPRY2 expression during human mesenchymal stem cell differentiation. J Cell Biochem.

[12] Wang C, Delogu S, Ho C (2012). Inactivation of Spry2 accelerates AKT-driven hepatocarcinogenesis via activation of MAPK and PKM2 pathways. J Hepatol.

[13] Kwak HJ, Kim YJ, Chun KR (2011). Downregulation of Spry2 by miR-21 triggers malignancy in human gliomas. Oncogene.

[14] Tay FC, Lim JK, Zhu H, Hin LC, Wang S (2014). Using artificial microRNA sponges to achieve microRNA loss-of-function in cancer cells. Adv Drug Deliv Rev.

[15] Wan L, Zhang L, Fan K, Wang J (2014). MiR-27b targets LIMK1 to inhibit growth and invasion of NSCLC cells. Mol Cell Biochem.

[16] Ye J, Wu X, Wu D (2013). miRNA-27b targets vascular endothelial growth factor C to inhibit tumor progression and angiogenesis in colorectal cancer. PLoS One.

[17] Ishteiwy RA, Ward TM, Dykxhoorn DM, Burnstein KL (2012). The microRNA -23b/-27b cluster suppresses the metastatic phenotype of castration-resistant prostate cancer cells. PLoS One.

[18] Lee JJ, Drakaki A, Iliopoulos D, Struhl K (2012). MiR-27b targets PPARgamma to inhibit growth, tumor progression and the inflammatory response in neuroblastoma cells. Oncogene.

[19] Jin L, Wessely O, Marcusson EG, Ivan C, Calin GA, Alahari SK (2013). Prooncogenic factors miR-23b and miR-27b are regulated by Her2/Neu, EGF, and TNF-α in breast cancer. Cancer Res.

[20] Li P, Tao L, Yang J (2013). Sprouty2 is associated with prognosis and suppresses cell proliferation and invasion in renal cell carcinoma. Urology.

[21] Rathmanner N, Haigl B, Vanas V, Doriguzzi A, Gsur A, Sutterlüty-Fall H (2013). Sprouty2 but not Sprouty4 is a potent inhibitor of cell proliferation and migration of osteosarcoma cells. FEBS Lett.

[22] Biyashev D, Veliceasa D, Topczewski J (2012). miR-27b controls venous specification and tip cell fate. Blood.

